# Preventing social defeat stress-induced behavioural and neurochemical alterations by repeated treatment with a mix of *Centella asiatica*, *Echinacea purpurea* and *Zingiber officinale* standardized extracts

**DOI:** 10.3389/fphar.2024.1439811

**Published:** 2024-08-26

**Authors:** Alessia Costa, Laura Micheli, Virginia Sordi, Clara Ciampi, Jacopo Lucci, Maria Beatrice Passani, Gustavo Provensi

**Affiliations:** ^1^ Section of Pharmacology and Toxicology, Department of Neuroscience, Psychology, Drug Research and Child Health (NEUROFARBA), University of Florence, Florence, Italy; ^2^ Pharmacology Unit, School of Pharmacy, University of Camerino, Camerino, Italy; ^3^ Bios-Therapy, Physiological Systems for Health S.p.A., Sansepolcro, Italy; ^4^ Aboca S.p.A. Società Agricola, Innovation and Medical Science Division, Sansepolcro, Italy; ^5^ Department of Health Sciences (DSS), University of Florence, Florence, Italy

**Keywords:** chronic social defeat stress, botanical drugs, cognition, pain, neuroinflammation, neurotrophins

## Abstract

**Background:** Prolonged exposure to stress is a risk factor for the onset of several disorders. Modern life is burdened by a pervasive prevalence of stress, which represents a major societal challenge requiring new therapeutic strategies. In this context, botanical drug-based therapies can have a paramount importance.

**Methods:** Here we studied the preventive effects of a repeated treatment (p.o. daily, 3 weeks) with a combination of Centella asiatica (200 mg/kg), Echinacea purpurea (20 mg/kg) and Zingiber officinale (150 mg/kg) standardized extracts, on the chronic social defeat stress (CSDS) deleterious outcomes. After 10 days of CSDS exposure, male mice’ performances were evaluated in paradigms relevant for social (social interaction test), emotional (tail suspension test), cognitive (novel object recognition) domains as well as for pain perception (cold plate and von Frey tests) and motor skills (rotarod). Mice were then sacrificed, the spinal cords, hippocampi and frontal cortices dissected and processed for RT-PCR analysis.

**Results:** Extracts mix treatment prevented stress-induced social aversion, memory impairment, mechanical and thermal allodynia and reduced behavioural despair independently of stress exposure. The treatment stimulated hippocampal and cortical BDNF and TrkB mRNA levels and counteracted stress-induced alterations in pro- (TNF-α, IL-1β and IL-6) and anti-inflammatory (IL4, IL10) cytokines expression in the same areas. It also modulated expression of pain related genes (GFAP and Slc1a3) in the spinal cord.

**Conclusion:** The treatment with the extracts mix obtained from *C. asiatica*, *E. purpurea* and *Z. officinale* may represent a promising strategy to promote resilience and prevent the deleterious effects induced by extended exposure to psychosocial stress.

## 1 Introduction

Stress can be defined as a process in which environmental demands strain an organism’s adaptive capacity (Cohen et al., 2016). Prolonged exposure to stress leads to a constellation of physiological, endocrine, immunological and behavioural alterations representing a major risk factor for the onset of several diseases, in particular psychiatric disorders such as anxiety and depression (Godoy et al., 2018). Modern day life is burdened by an insidious increasing prevalence of psychological stress, representing a major pervasive societal challenge that urgently requires new therapeutic strategies. Promoting resilience to stress may prevent the development of stress-induced psychiatric disorders, and in this context, therapies based on natural products may acquire paramount importance since they are perceived by the patients as safer alternatives to classical pharmacotherapy (Yeung et al., 2018). Many medicinal plants have long been used in folk medicine for treating central nervous system-related pathologies. The effects of a broad range of plants have been confirmed in animal models and for some of them also in clinical trials (Moragrega and Ríos, 2021; Moragrega and Ríos, 2022). Medicinal plants can offer a valid approach to tackling stress-related disorders since they are characterized by a complex mixture of bioactive metabolites which can antagonize the multifactorial and intricate mechanisms of the disease (Colalto, 2018). *Centella asiatica, Echinacea purpurea* and *Zingiber officinale* are plants rich in bioactive molecules with multiple pharmacological effects.

*Centella asiatica* is a plant traditionally used in Ayurvedic medicine as sedative and anxiolytic (Zhang, 2004). Pharmacological studies confirmed such effects (Chanana and Kumar, 2016) and revealed new ones such as anti-hyperalgesic properties in a rat model of osteoarthritis (Micheli et al., 2020b), and procognitive actions (Sbrini et al., 2020). The most conspicuous group of bioactive metabolites isolated from *C. asiatica,* are the triterpenoids, including madecassoside, asiaticoside, asiatic acid and madecassic acid (Mando et al., 2024). *Echinacea* species are medicinal plants of the Asteraceae family native from United States, Canada, Russia and Australia among which *E. purpurea* is one of best known and used from a medical point of view (Burlou-Nagy et al., 2022). This species is considered a valid source of anti-inflammatory metabolites particularly active to boost the immune system (Li et al., 2020; Sharifi-Rad et al., 2018). *Echinacea purpurea* is traditionally used as pain reliever. Recent pharmacological studies confirmed its analgesic properties, which are related to the presence of alkamides, caffeic acid derivatives and polysaccharides (Barnes et al., 2005; Micheli et al., 2023). Among the botanical drugs with potential neuroprotective and anti-inflammatory properties, much attention has been focused on extracts obtained from the Zingiberaceae family. Pharmacologically speaking, *Z. officinale* extracts, rich in gingerols, paradols, and shogaols, shares similarities with non-steroidal anti-inflammatory drugs in inhibiting the activity of cyclooxygenase-2 (COX-2) and lipoxygenase (LOX) enzymes and prostaglandin synthesis (Grzanna et al., 2005). Consistently, the capacity of *Z. officinale* extracts of reducing the production of inflammatory mediators, such as IL-1β, and TNF-α, were demonstrated in *in vitro* (Shen et al., 2003; Shen et al., 2005) and *in vivo* models (Oliveira et al., 2019; Micheli et al., 2022). Several studies evidenced ginger extracts-induced neuroprotective effects, that seem to be related to their anti-oxidative and anti-inflammatory properties as well as to the upregulation of neurotrophins (ALmohaimeed et al., 2021).

We recently reported the beneficial effects of a treatment with the standardized extracts mix obtained from *C. asiatica, E. purpurea* and *Z. officinale* in a model of lipopolysaccharide (LPS)-induced neuroinflammation in mice (Micheli et al., 2022). Here we aimed to expand these observations by assessing the efficacy of the chronic treatment with the same mix of extracts in preventing the alterations induced by chronic social defeat stress (CSDS) in adult mice. This procedure induces behavioural and neurochemical alterations characterized by social avoidance, memory impairment (Golden et al., 2011; Costa et al., 2022), immune system responses in the brain (Hodes et al., 2014; Shimo et al., 2023), impaired blood brain barrier integrity (Dudek et al., 2020) and may affect pain perception, as reviewed in (Vachon-Presseau, 2018). A series of tests were therefore performed to evaluate the efficacy of extracts mix to prevent CSDS-related behavioural outcomes, along with *ex vivo* assessment of neurotrophins, pro- and anti-inflammatory cytokines and blood-brain barrier integrity markers expression at the transcriptional level in the spinal cord, hippocampus and prefrontal cortex.

## 2 Materials and methods

### 2.1 Animals

Adult male C57bl/6 (20–25 g, 8–9 weeks old, *intruders*) and CD1 mice (35–45 g, 10–13 weeks old, *residents*) purchased from Charles River Laboratories (Milan, Italy) were used in this study. These strains are the most commonly *intruder/submissive:resident/aggressive* pair used in CSDS protocols (Golden et al., 2011). They were housed at the animal facility of the Centro di Servizi per la Stabulazione di Animali da Laboratorio (CeSAL) of the University of Florence, in humidity and temperature-controlled rooms (22°C ± 2°C) with free access to food (4RF21; Mucedola s.r.l., Italy) and water, and kept on a 12-h light/dark cycle (lights start at 8:00 a.m.). After arrival, animals were allowed 1 week to acclimatize to the housing conditions and human contact before the beginning of the experiments. All the experiments were performed between 9:00 a.m. and 2:00 p.m.

Housing and experimental procedures were conducted in accordance with the Council Directive of the European Community (2010/63/EU) and the Italian Decreto Legislativo 26 (13/03/2014) regarding the protection of animals used for scientific purposes, approved by the Animal Care Committee of the University of Florence and Italian Ministry of Health (678-2021-PR prot. 17E9C.235) and supervised by a veterinarian. Every effort was made to minimize animal suffering and to reduce the number of animals used, complying with the 3R principle. Male mice were used to reduce within-group variability due to hormonal fluctuations during oestrous cycle in female mice.

### 2.2 Plant material, extracts preparation and standardization

The following botanical drugs were used in this study: *C. asiatica* (L.) Urb [Apiaceae, *C. asiatica* (L.). Urb., herba], *E. purpurea* (L.) Moench (Asteraceae, *Echinaceae purpureae herba*) and *Z. officinale* Roescoe (Zingiberaceae; *Zingiber officinale* Roscoe, rhizome). The botanical indentification, extracts production and quantification of main metabolites were performed at Aboca S.p.A. (Sansepolcro, Italy). The extracts were prepared following the protocols described previously (Micheli et al., 2020b; Micheli et al., 2022). The preparation and characterization of the extracts studied in our manuscript fulfill all the requirements established by the Consensus-based reporting guidelines for Phytochemical Characterisation of Medicinal Plant extracts (ConPhyMP) (see Supplementary Tables)*.* Briefly, dried *Z. officinale* rhizoma was subjected to extraction by using 30% ethanol (ethanol: water, 30:70 v/v) for 8 h at 50°C and filtered to remove solid exhausted material. The resulting clarified extract was concentrated by ethanol evaporation under vacuum, until reaching the concentration ratio of 10:1 (v:v, initial alcoholic extract volume compared to the volume after the evaporation step), and then freeze-dried for 72 h. Final drug-extract ratio (DER) = 10.85. Dried flowering tops of *E. purpurea* were subjected to extraction by using 45% ethanol (ethanol: water, 45:55 v/v) for 8 h at 50°C and filtered to remove solid exhausted material. The resulting clarified extract was concentrated by ethanol evaporation under vacuum, until reaching the concentration ratio of 8:1 (v:v, initial volume of alcoholic extract compared to the volume after evaporation step), and then freeze-dried for 72 h. DER = 6.3. Dried *C. asiatica* leaves were subjected to extraction with ethanol 70% (ethanol:water 70:30 v/v) for 8 h at 50°C and filtered to remove solid exhausted material. The resulting clarified extract was concentrated by ethanol evaporation under vacuum, until reaching the concentration ratio of 7:1 (v:v, initial alcoholic extract volume compared to the volume after the evaporation step), then freeze-dried for 72 h. DER = 4.3. The resulting extracts were stored at 4°C until use, away from light and humidity. The characterization of all extracts was also performed by Aboca S.p.A. following the USP Pharmacopoeia Monographies using different previously reported chromatographic methods (Micheli et al., 2020b; Micheli et al., 2022). *Centella asiatica* extract*:* Triterpenes, total, 5.01% (Asiatic Acid, 0.09%; Madecassic Acid, 0.66%; Asiaticoside, 1.96%, Madecassoside, 2.30%); *E. purpurea* extract: Phenols, total 5.95% (Chlorogenic acid 0.04%, Caftaric acid, 2.08%, Cicoric acid 3.83%); *Z.officinale* extract*:* Gingerols, totals 3.107% (6-Gingerol, 2.539%; 8-Gingerol 0.339%, 10-Gingerol 0.229%), Shogaols, totals, 0.647%.

### 2.3 Treatments

The combination of extracts obtained from *C. asiatica* (200 mg/kg), *E. purpurea* (20 mg/kg) and *Z. officinale* (150 mg/kg) were suspended in 1% carboxymethylcellulose sodium salt (CMC; Sigma-Aldrich, Milan, Italy) and orally administered (by gavage) once daily. The doses were chosen based on our previous study reporting their efficacy in an LPS-induced neuroinflammation mouse model (Micheli et al., 2022). To evaluate a potential preventive effect induced by extracts mix, treatments started 21 days before stress exposure and lasted until the end of behavioural experiments on day 37. Control animals received an equal volume of vehicle. Both vehicle and extracts solutions were freshly prepared on each test day and administered during the light-phase (between 4:00 and 6:00 p.m.) to avoid confounding factors regarding putative acute effects on the behavioural tests (performed between 9:00 a.m. and 2:00 p.m.).

### 2.4 Experimental design

**FIGURE 1:**
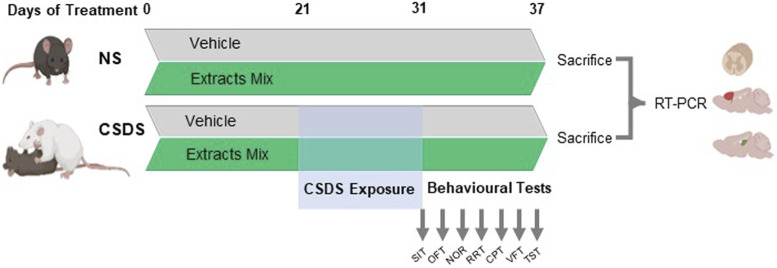
Experimental timeline. C57BL/6 mice were treated with vehicle or the combination of extracts obtained from *Centella asiatica* (200 mg/kg, p.o.), *Echinacea purpurea* (20 mg/kg, p.o.) and *Zingiber officinale* (150 mg/kg, p.o.) once daily. After 21 days of treatment, mice were submitted to a 10-days chronic social defeat stress protocol (CSDS). Immediately after the end of stress exposure, animals’ behaviour was evaluated using a battery of tests in the following order: social interaction test (SIT), open field (OFT), novel object recognition (NOR), rotarod test (RRT), cold plate test (CPT), von Frey test (VFT) and tail suspension test (TST). The day following the last test, animals were sacrificed, the spinal cord, hippocampi and frontal cortices were dissected and processed for gene expression analysis at transcriptional level by real-time polymerase chain reaction (RT-PCR).

C57BL/6J experimental mice were randomly assigned to the following experimental groups (6–8 animals by group): a control group of non-stressed animals treated with vehicle (NS-Vehicle), a group of non-stressed animals treated with the extracts mix (NS-Extracts Mix), a group of stressed mice treated with vehicle (CSDS-Vehicle) and finally, a group of stressed animals receiving the extracts mix daily (CSDS-Extracts Mix). CSDS procedure started on the 21st day of treatment and lasted for 10 days. To evaluate CSDS-related outcomes, animals’ behavioural repertoire was assessed using a battery of tests comprehensive of several domains potentially affected by stress. Behavioural assessment was conducted from day 31 to 37. The tasks were performed in separated days, starting with the least stressful/invasive procedure, in the following sequence: social interaction test, open field, novel object recognition, rotarod, von Frey test, cold plate test and tail suspension test. Twenty-four hours after the last behavioural test, animals were sacrificed, and the tissues were collected and stored at −80°C for the *ex vivo* analyses. An overview of the experimental design is shown in Figure 1.

### 2.5 Chronic social defeat stress (CSDS)

The CSDS paradigm was performed as previously described (Rani et al., 2021; Costa et al., 2022). Prior to the experiments, CD1 mice were singly housed and screened for aggressive behaviour according to (Golden et al., 2011). Only those reaching the criterion, defined by demonstrating at least one successful act of aggression during two consecutive days toward another CD1 intruder mouse, were selected as aggressive-residents and used in the CSDS paradigm. C57BL/6 mice randomly allocated to the experimental stressed groups were singly housed for 4 days prior to the stress exposure. Briefly, the CSDS procedure consisted in the introduction of an experimental C57BL/6 mouse in the home cage of a CD1 aggressor until the occurrence of an aggression. Mice were then separated by a transparent, perforated Plexiglas separator wall allowing visual and olfactory exposure for 2 h. After that the separator was removed, allowing a second attack occurring within 1 min. The procedure was repeated daily for 10 consecutive days (days 21–30), at different timing during the day and changing the CD1 aggressor every day to avoid habituation. The stress protocol included overcrowding sessions that consisted of 8 experimental mice placed together in a standard holding cage (33 cm × 15 cm × 13 cm) for 24 h (days 23–24, 28–29) with diet and water available *ad libitum.* Non-stressed mice were left undisturbed in their home cages housed with other non-stressed mice (four mice per cage) being manipulated only for gavage treatment and body-weight measurement.

### 2.6 Social interaction test (SIT)

SITs were conducted 24 h after the last social defeat session, in an acrylic Plexiglass arena (41 cm × 32 cm x 40 cm) adopting the protocol described in (Golden et al., 2011; Rani et al., 2021; Costa et al., 2022). The SIT protocol is articulated in two sessions: target absent (−) and target present (+) lasting 150s each, with an inter-trial interval of 60s between sessions. In the first session (−) C57BL/6 subject mice were gently placed in the centre of the arena containing an empty wire-mesh box (7.5 cm length, 9.5 cm width) and left free to explore. During the second session (+) C57BL/6 mice were placed in the same arena, this time with a CD1 aggressive mouse inside a wire-mesh container and were again allowed to explore freely. During the inter-trial interval, experimental C57BL/6 mice were placed back in their home-cages. After each session, the arena and the box were cleaned with an ethanol solution (30% v/v) to prevent odour/taste bias. The trials were videotaped, and the time C57BL/6 mice spent in the interaction zone (5 cm around the wire mesh cage) actively exploring the box, was recorded offline by an experienced observer blinded to the experimental groups assignment. Exploration was defined as sniffing or touching the cage with the nose and/or forepaws. A social interaction index was calculated as the ratio between the time spent in the interaction zone during − and + sessions, following the equation:

$$Social\;Interaction\;Ratio\;\left(SI\;ratio\right)=\frac{time\;exploring\;SI\;zone\;during+session\;\left(t+\right)}{time\;exploring\;SI\;zone\;during-session\;\left(t-\right)}$$

### 2.7 Open field test (OFT)

Mice locomotor activity was assessed in an open field arena (44 × 44 × 30 cm) constructed in grey Plexiglass. Each C57BL/6 mouse was placed in the centre of the arena and allowed to freely explore it for 10 min. The arena was cleaned with a solution of 30% ethanol (v/v) between trials to remove possible scent cues left by the animals. The experiments were recorded with a camera located on the ceiling above the arena. Using the ANY-Maze (version 7.13, SoeltingCo^®^) video tracking system software a virtual zone (22 cm × 22 cm) was delimited in the centre of the arena and the distance travelled by each animal in either central and peripheral zones were registered (Rani et al., 2021; Costa et al., 2022).

### 2.8 Novel object recognition test (NOR)

The NOR was performed following the detailed experimental procedure previously published (Rani et al., 2021; Costa et al., 2022; Provensi et al., 2022). The protocol involved three sessions: habituation, acquisition and retention test. Habituation consisted of a 10 min session where the animals were left to freely explore the empty arena (44 cm × 44 cm × 30 cm) constructed in grey Plexiglass. Twenty-four hours later, each C57BL/6 mouse was placed in the same position facing the same direction into the test arena in the presence of two identical objects (plastic shapes such as cubes, cylinders or pyramids 8 cm high) and allowed to explore it for 5 min. To evaluate the impact of stress and treatments on short-term memory, the retention test session was performed 1 h later. During this session, mice were again placed in the test arena for 5 min in the presence of one familiar object and a novel one. The position of the new object (left/right) was randomized to prevent bias from order or place preference. Animals’ behaviour during all sessions was videotaped and the time spent actively exploring each object was recorded by an experienced researcher unaware of the experimental groups. Exploration was defined as sniffing or touching the objects with the nose and/or forepaws. Sitting on or turning around the objects was not considered exploratory behaviour. Each animal was subjected to the procedure separately and care was taken to remove any olfactory/taste cues by cleaning carefully the arena and test objects between trials with an ethanol solution (30% v/v). Mice were placed in their home cages between trials. The final data is expressed as the percentage of time exploring the familiar and new objects during the retention test. The raw exploration data at the retention test was also used to calculate individual discrimination indexes (DIs) according to the follow equation:

$$Discrimination\;Index\;\left(DI\right)=\frac{time\;esploring\;novel\;\left(tN\right)-\;time\;exploring\;familiar\;\left(tF\right)}{total\;exploration\;time\;\left(tN+tF\right)}$$

### 2.9 Tail suspension test (TST)

The TST, which is widely used to assess drug-induced antidepressant-like effects, was carried out as previously described (Munari et al., 2015; Costa et al., 2018). Briefly, each C57BL/6 mouse was suspended by the tail to a horizontal bar at approximately 30 cm above the floor using adhesive tape (approximately 2 cm from the tip of the tail) for 6 min. The sessions were videotaped and analysed by an experimenter unaware of the experimental groups. The parameter recorded was the number of seconds animals spent immobile during the last 4 min of each session. Mice were considered immobile only when they hung passively and completely motionless.

### 2.10 Cold plate test

Thermal allodynia was assessed using the cold plate test (Ugo Basile, Varese, Italy). With minimal animal–handler interaction, mice were taken from home cages and placed onto the surface of the cold plate maintained at a constant temperature of 4°C ± 1°C. Ambulation was restricted by a cylindrical Plexiglas chamber (diameter, 10 cm; height, 15 cm) with an open top. A timer controlled by a foot peddle was used to monitor timing response latency from the moment the mouse was placed onto the cold plate. Pain-related behaviour (licking of the hind paw) was observed, and the time (seconds) of the first sign was recorded. The cutoff time of paw lifting or licking latency was set at 30 s (Micheli et al., 2022; Micheli et al., 2020a).

### 2.11 von Frey test

An electronic von Frey hair unit (Ugo Basile, Varese, Italy) was used to test mechanical allodynia. The animals were placed in 20 cm × 20 cm Plexiglas boxes equipped with a metallic mesh floor, 20 cm above the bench. A habituation of 15 min was allowed before the test. The withdrawal threshold was evaluated by applying force ranging from 0 to 5 g with a 0.2 g accuracy. Punctuate stimulus was applied to the mid-plantar area of each anterior paw from below the meshy floor through a plastic tip, and the withdrawal threshold was automatically displayed on the screen. The paw sensitivity threshold was defined as the minimum pressure required to elicit a robust and immediate withdrawal reflex of the paw. Voluntary movements associated with locomotion were not taken as a withdrawal response. Stimuli were applied on each anterior paw with an interval of 5 s. The measurement was repeated five times, and the final value was obtained by averaging the five measures (Micheli et al., 2022; Micheli et al., 2021).

### 2.12 Rotarod test

The apparatus consisted of a base platform and a rotating rod with a diameter of 3 cm and a non-slippery surface. The rod was placed at a height of 15 cm from the base. The rod, 30 cm in length, was divided into five equal sections by six disks. Thus, up to five mice were tested simultaneously on the apparatus, with the rotating speed of the rod at 16 revolutions per minute. The integrity of motor coordination was assessed based on the number of falls from the rod in 10 min (Micheli et al., 2022).

### 2.13 Real-time PCR of gene expression in the spinal cord, hippocampus and frontal cortex

The hippocampi, frontal cortices and thoracic portions of the spinal cord were rapidly removed and stored at −80°C. RNA was extracted using TRIzol reagent (Invitrogen, Life Technologies™ Italia). RNA concentrations were determined using a Nanodrop ND-1000 (Labtech, Inc.). cDNA was synthesized with Reliance Select cDNA Synthesis Kit (Bio-Rad Laboratories, Inc.) according to the manufacturer’s instructions. Briefly, 1 µg of total RNA mixed with DNase Buffer and DNase (both from Bio-Rad Laboratories, Inc.) was incubated at 25°C for 5 min and at 75°C for 5 min 4 μL Reliance Buffer, 2 µL Random Primer mix and 1 µL of Reliance Reverse Trascriptase were added to each sample and incubated at 50°C for 20 min and at 95°C for 1 min. All RNA samples were synchronously reverse transcribed to minimize interassay variations related to the reverse transcription reaction.

RT-PCR was performed using SsoAdvanced Universal SYBR^®^ Green Supermix (Bio-Rad Laboratories, Inc.) following the default thermocycler program: 2 min of preincubation at 95°C followed by 40 cycles for 5 s at 95°C, 30 s at 60°C and 5 s/step at 65°C–95°C with a 0.5°C increments. RT-PCR reactions were carried out in 10 μL volumes in a 96-well plate (Applied Biosystems™, London, United Kingdom) containing 2 μL RNAse-free water, 1 μL of primer (Bio-Rad Laboratories, Inc.), and 5 μL Universal SYBR^®^ Green Supermix (Bio-Rad Laboratories, Inc.) plus 2 μL of cDNA sample (10 ng/μL).

The following validated primers purchased from Bio-Rad Laboratories, Inc., were used to detect *Bdnf* (qMmuCED0050333), *Ntrk2* (qMmuCID0016820), *Tnf* (qMmuCED0004141), *Il1b* (qMmuCID0005641), *Il6* (qMmuCID0005613), *Il4* (qMmuCID0006552), *Il10* (qMmuCID0015452), *Aif1* (qMmuCED0025128)*, Gfap* (qMmuCIP0032231), *Serpina3n (*qMmuCID0024737), *Scl1a2* (qMmuCIP0031555), *Scl1a3* (qMmuCIP0030473)*, Ocln* (qMmuCID0005446), *Cldn5* (qMmuCED0001017), *Marveld2* (qMmuCID0008476), *Tjp1* (qMmuCID0005277), *Cdh5* (qMmuCID0005343), and *Pecam1* (qMmuCID0005317). The differential expression of the transcripts was normalized on the housekeeping gene *Gapdh* (QT01658692, QIAGEN Sciences, LLC). The data of real time PCR are expressed as Fold Change Quantification, each experiment was repeated in triplicate, and quantitative PCR analysis was analyzed with 2^−▵▵Ct^-method (Livak and Schmittgen, 2001).

### 2.14 Statistical analysis

The data were analysed using Graphpad Software (version 10.2.1). Statistical significance was determined using a Two-way or Three-way ANOVA, as appropriate for the variables analysed in each experimental set, followed by Bonferroni’s multiple comparison *post hoc* test. The level of significance was set to *P* < 0.05. Outliers were identified and excluded from each experimental set using the ROUT method (Motulsky and Brown, 2006). Data shown in figures are expressed as individual points for each animal (aligned dot plot). Bar graphs represent means ± standard error of the mean (S.E.M).

## 3 Results

### 3.1 Chronic treatment with extracts mix prevented CSDS-induced social avoidance

**FIGURE 2:**
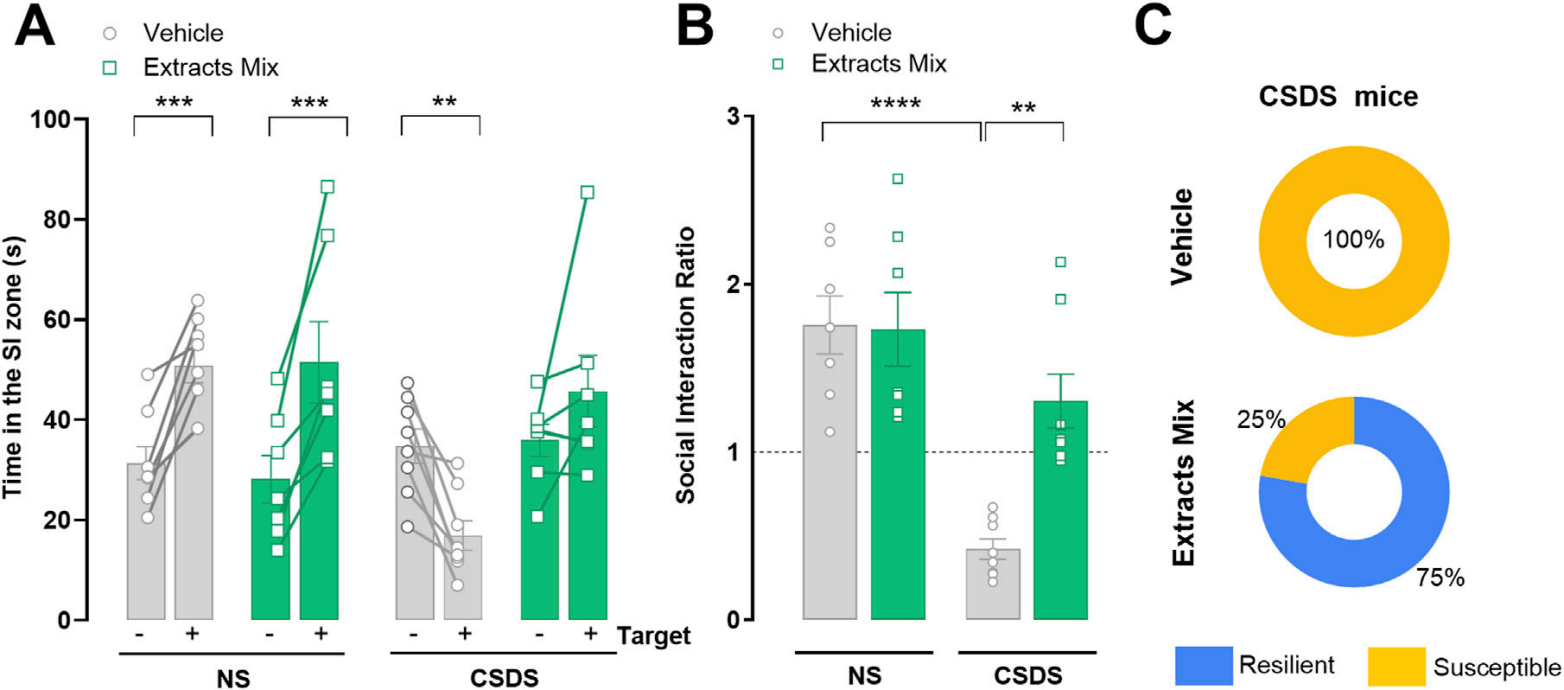
Effect of chronic treatment with extracts mix on stress-induced social avoidance. **(A)** Time spent in the social interaction (SI) zone during target absent (−) and target present (+) sessions. **P < 0.01 ***P < 0.001, Three-Way ANOVA and Bonferroni’s test. **(B)** SI Ratios calculated for each animal. **P < 0.01 ****P < 0.0001, Two-Way ANOVA and Bonferroni’s test **(C)** Percentage of animals exposed to CSDS classified as susceptible (SI Ratio <1) or resilient (SI Ratio >1). N = 7–8 animals per experimental group.

Following 10 days of CSDS exposure, animals’ susceptibility to stress was evaluated by comparing the time spent in the interaction zone during target absent (−) and target present (+) sessions of the SIT (Figure 2). As expected, non-stressed animals spent more time in the interaction zone when the target was present, independently of the treatment (*P* < 0.001). Vehicle-treated stressed animals, instead, spent less time in the interaction zone during the (+) session (*P* < 0.01). Treatment with extracts mix prevented such effect, as revealed by the longer time spent by the animals in the interaction zone when a CD1 mouse was present (Figure 2A). In keeping with these results, a significant reduction of SI ratios emerged for the group of stressed animals treated with vehicle (*P* < 0.0001), which was prevented by the chronic treatment with the extracts mix (*P* < 0.01) (Figure 2B). Analysing SI ratios individually (Figure 2C), we found that all animals allocated to the CSDS-Vehicle group showed a SI <1, confirming that our CSDS protocol was sufficient to generate a large population of stress-susceptible mice. Regarding the group of stressed animals treated with extracts mix, 6 out of 8 stressed animals had a SI ratio >1 (corresponding to 75% of the group), a phenotype classified as stress resilient. For the two remaining animals, the calculated SI ratios were 0.98 and 0.94, very close, but still below the unit, therefore they were classified as stress-susceptible phenotypes (Golden et al., 2011).

### 3.2 Chronic treatment with extracts mix attenuated behavioural despair and prevented stress-induced memory impairment

**FIGURE 3:**
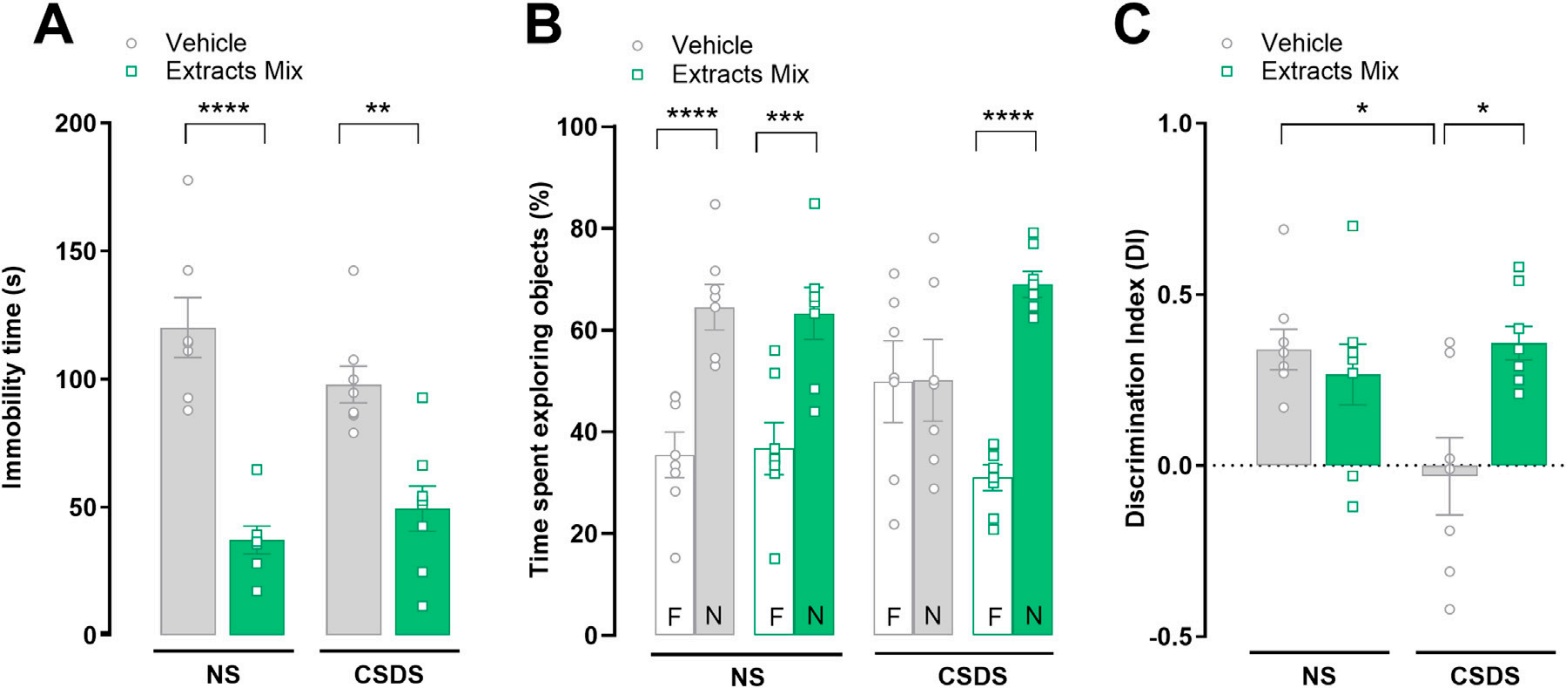
Effect of chronic treatment with extracts mix on behavioural despair and stress-induced short-term memory impairment. **(A)** Immobility time, measured in seconds, in the tail suspension test. **P < 0.01 ****P < 0.0001, Two-Way ANOVA and Bonferroni’s test. **(B)** Percentage of time spent exploring the familiar (F) or novel (N) objects during the retention test, measured 1 h after acquisition, in the novel object recognition paradigm. ***P < 0.001, ****P < 0.0001, Three-Way ANOVA and Bonferroni’s test. N = 7–8 animals per experimental group. **(C)** Discrimination Index (DI) calculated following the equation DI = (time spent exploring N–time exploring F)/total time spent exploring objects. *P < 0.05 Two-Way ANOVA and Bonferroni’s test. N = 7–8 animals per experimental group.

As shown in Figure 3A chronic treatment with extracts mix significantly decreased the immobility time measured in the tail suspension test, of both stressed (*P* < 0.01) and non-stressed (*P* < 0.001) mice. Next, the impact of stress and treatments on cognition was evaluated with the NOR test. Non-stressed animals, regardless of treatment, recognized the familiar object and thus explored the new object for a longer time (*P* < 0.001). Exposure to CSDS had a detrimental effect on recognition memory in vehicle-treated mice, as they did not discriminate between the two objects, confirmed by the significantly lower DI as compared to the NS-Veh group (Figure 3C, *P* < 0.005). Chronic treatment with extracts mix prevented stress-induced amnesic effects as revealed by the increased time mice spent exploring the new object (Figure 3B, *P* < 0.001), as well as by the DI which was significantly higher than the value observed in CSDS-Veh animals (Figure 3C, *P* < 0.005).

### 3.3 Chronic treatment with extracts mix reduced stress-induced thermal and mechanical allodynia

**FIGURE 4:**
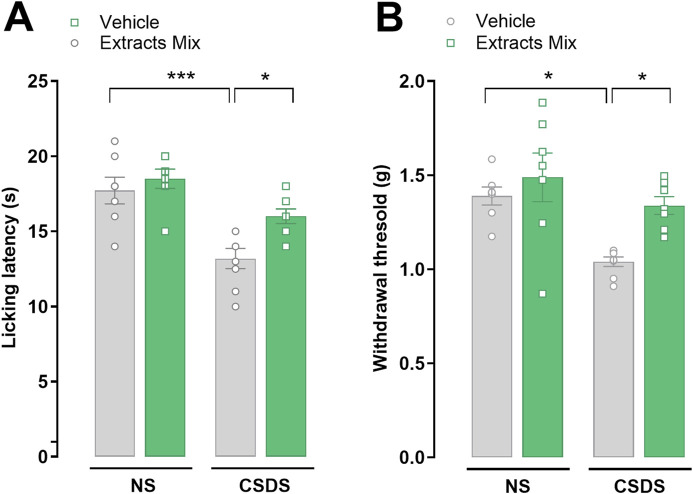
Effects of chronic treatment with extracts on stress-induced thermal and mechanical allodynia. Pain threshold alterations evoked by a thermal and mechanical non-noxious stimuli were evaluated using the **(A)** cold plate and **(B)** the von Frey tests, respectively. *P < 0.05 ***P < 0.001, Two-Way ANOVA and Bonferroni’s test. N = 6–8 animals per experimental group.

Pain threshold alterations were evaluated using the cold plate and the von Frey tests. As illustrated in Figure 4A 10 days of CSDS exposure significantly reduced mice licking latency in comparison to non-stressed animals (*P* < 0.001) in the cold plate test. Chronic treatment with the extracts mix significantly increased the time spent by the animals on the cold surface (*P* < 0.05) demonstrating that the extracts mix protected against CSDS-induced thermal allodynia. Similar results were obtained when mice were challenged using a non-noxious mechanical stimulus (von Frey test; Figure 4B). Chronic treatment with extracts mix prevented the development of mechanical allodynia evoked by stress exposure (*P* < 0.05) as it increased paw withdrawal threshold up to the values recorded in the non-stressed group. To note, the repeated administration of the extracts mix to non-stressed mice did not affect pain threshold (Figure 4).

### 3.4 Effects of CSDS and extracts mix on food intake, body weight gain and locomotor activity

**FIGURE 5:**
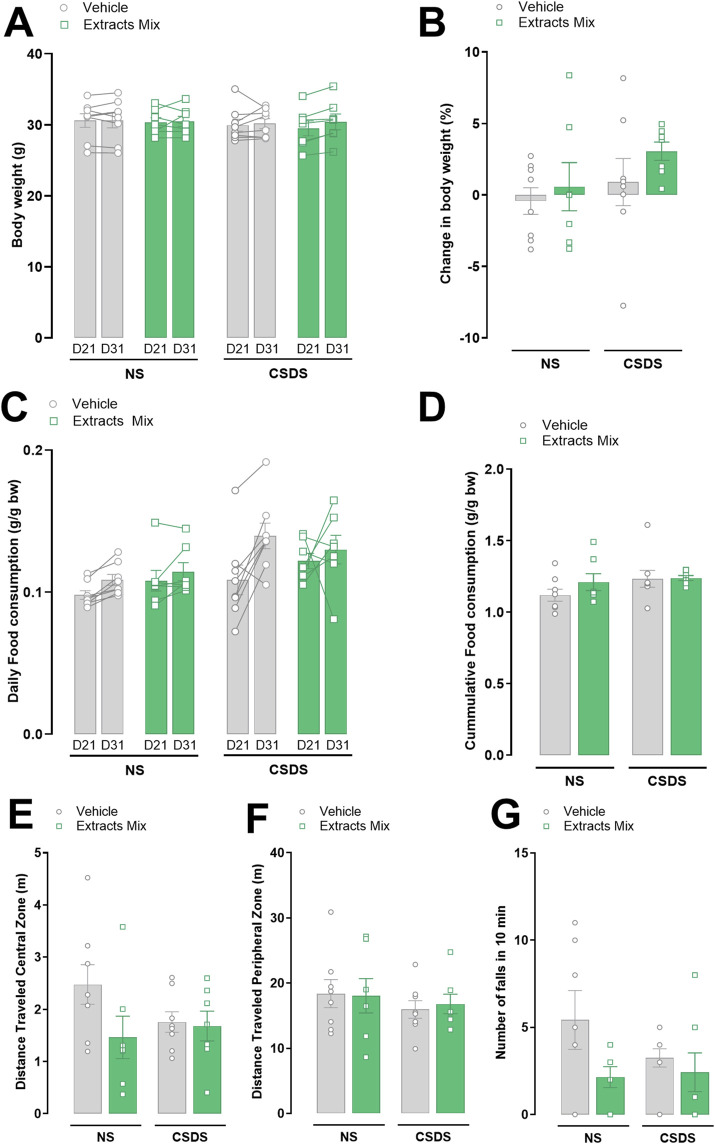
Effects of stress and chronic treatment with extracts mix on food consumption, body weight and locomotor activity. Body weight **(A,B)** and food consumption **(C,D)** measured before (D21) and after (D31) stress exposure. Distance travelled in the center **(E)** and periphery **(F)** of an Open Field arena in a 10 min test. Number of falls **(G)** registered in the rotarod during a 10 min session. No differences between groups were detected using Two-Way ANOVA and Bonferroni’s test. N = 6–8 animals per experimental group.

Locomotion, measured as the distance travelled in both the central and peripheral zones of an open field arena, was not different between experimental groups (Figures 5E, F). No differences emerged also regarding the number of falls in a 10 min rotarod session (Figure 5G). These data indicate that the effects observed in the previous sessions (stress and treatment) cannot be ascribed to locomotor alterations or motor incoordination.

Food consumption and body weight were measured daily. Figures 5A–D shows the data after 21st and 31st days of treatment with extracts mix or vehicle, i.e., before and after animals’ exposure to the 10-day CSDS. Neither stress nor chronic treatment with extracts mix altered the amount of eaten food (Figures 5C, D) and body weight gain (Figures 5A, B).

### 3.5 Chronic treatment with extracts reduced mRNA expression of pain-related markers altered by stress in the spinal cord

**FIGURE 6:**
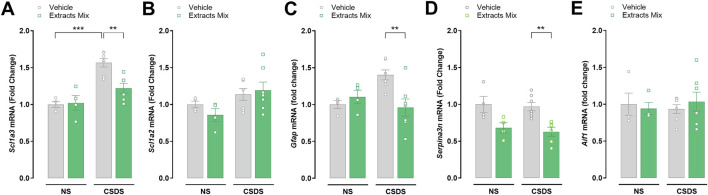
Effects of treatment with extracts mix on transcription of pain-related markers in the spinal cord. Fold changes in mRNA expression of *Scl1a3* **(A)**, *Scl1a2* **(B)**, *Gfap* **(C)**, *Serpina3n* **(D)** and *Aif1* **(E)** genes relative to the control group determined by RT-PCR. **P < 0.01 ***P < 0.001, Two-Way ANOVA and Bonferroni’s test. N = 4–7 animals per experimental group.

The expression of pain-related genes was evaluated by RT-PCR analysis in spinal cord samples of non-stressed and stressed animals treated with the extracts mix or vehicle (Figure 6). CSDS exposure significantly increased the mRNA expression of *Scl1a3* (*P* < 0.001) in comparison to non-stressed animals and we observed a trend increase in *Gfap* expression (*P* = 0.0827), which did not reach statistical significance. The daily repeated treatment with the extracts mix prevented these changes (*P* < 0.01) without modifying the expression of the same genes in non-stressed animals (Figures 6A, C). Moreover, the extracts mix significantly down-modulated the expression of *Serpina3n* (*P* < 0.01; Figure 6D) in comparison to the CSDS-Veh group without affecting the mRNA expression of non-stressed mice. Spinal cord mRNA expression of *Scl1a2* (Figure 6B) and *Aif1* (Figure 6E) were not significantly altered by CSDS exposure nor by the extracts mix treatment.

### 3.6 Chronic treatment with extracts mix prevented CSDS-induced neuroinflammation

**FIGURE 7:**
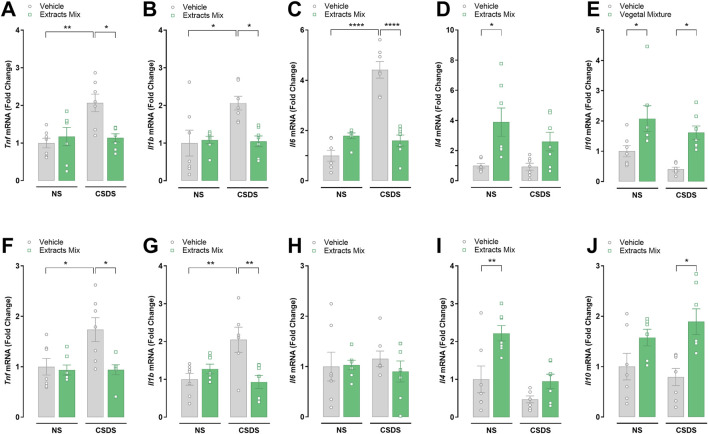
Effects of chronic treatment with extracts mix on stress-induced alterations in hippocampal **(A–E)** and cortical **(F–J)** cytokines mRNA expression. Fold changes in mRNA expression of *Tnf* **(A, F)**, *Il1b* **(B, G)**, *Il6* **(C, H)**, *Il4* **(D, I)** and *Il10* **(E, J)** genes relative to the control group determined by RT-PCR. *P < 0.05 **P < 0.01 ****P < 0.0001, Two-Way ANOVA and Bonferroni’s test. N = 7–8 animals per experimental group.

The alterations in the expression of several inflammatory mediators were determined at the transcriptional level by RT-PCR. Significant differences emerged among groups when analysing mRNA expression in hippocampal samples (Figures 7A–E). Specifically, CSDS exposure significantly increased the hippocampal expression of the pro-inflammatory cytokines TNF-α (Figure 7A, *P* < 0.01), IL-1β (Figure 7B, *P* < 0.05) and IL-6 (Figure 7C, *P* < 0.0001), which was completely prevented by chronic treatment with extracts mix (Figures 7A–C, *P* < 0.05, *P* < 0.05 and *P* < 0.0001, respectively). Regarding the anti-inflammatory cytokines, IL-4 mRNA expression was increased following extracts treatment reaching statistical significance only in the group of non-stressed animals (Figure 7D, *P* > 0.05), whereas a significant increase of IL-10 mRNA levels (Figure 7E, *P* < 0.05) was observed in animals chronically treated with the extracts mix, independently of the stress exposure.

The results emerging from the analysis of cytokines mRNA expression in cortical samples (Figures 7F–J) are largely similar to those observed in the hippocampus, except for the lack of IL-6 increase in the stressed *vs* non-stressed animals treated with vehicle, and IL-10 expression in NS-Extracts mix group compared to NS-Veh group, that did not reach statistical significance.

### 3.7 Effects of CSDS and extracts mix on mRNA expression of BBB integrity-related markers

**FIGURE 8:**
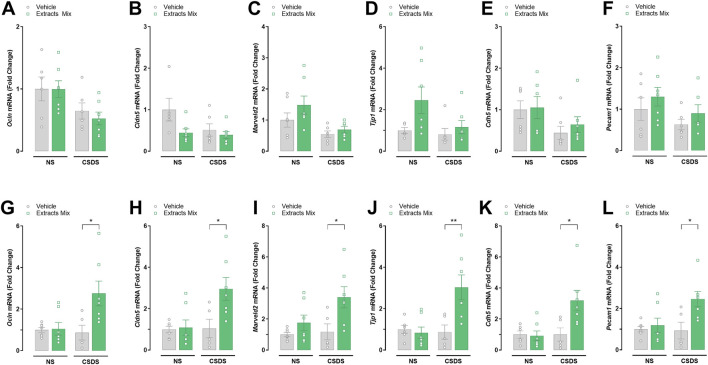
Effects of treatment with extracts mix on transcription of blood-brain barrier integrity-related markers in hippocampal **(A–F)** and cortical **(G–L)** samples. Fold changes in mRNA expression of *Ocln* **(A, G)**, *Cldn5* **(B, H)**, *Marveld2* **(C, I)**, Tjp1 **(D, J)**, *Cdn5* **(E, K)** and *Pecam1* **(F, L)** genes relative to the control group determined by RT-PCR. *P < 0.05, Two-Way ANOVA and Bonferroni’s test. N = 6–8 animals per experimental group.

We determined the expression, at the transcriptional level, of a series of tight junction and adherens proteins (paracellular proteins) in hippocampal (Figures 8A–F) and cortical (Figures 8G–L) samples. Significant increases in the mRNA transcripts for *Ocln* (*P* < 0.05), *Cldn5* (*P* < 0.05), *Marveld2* (*P* < 0.05), *Tjp1* (*P* < 0.05), *Cdh5* (*P* < 0.05) and *Pecam1* (*P* < 0.05) were found in the frontal cortex of stressed animals treated with extracts mix when compared to stressed animals receiving vehicle. No differences among groups were detected in hippocampal samples.

### 3.8 Chronic treatment with extracts mix increased hippocampal and cortical BDNF and TrkB mRNA expression

**FIGURE 9:**
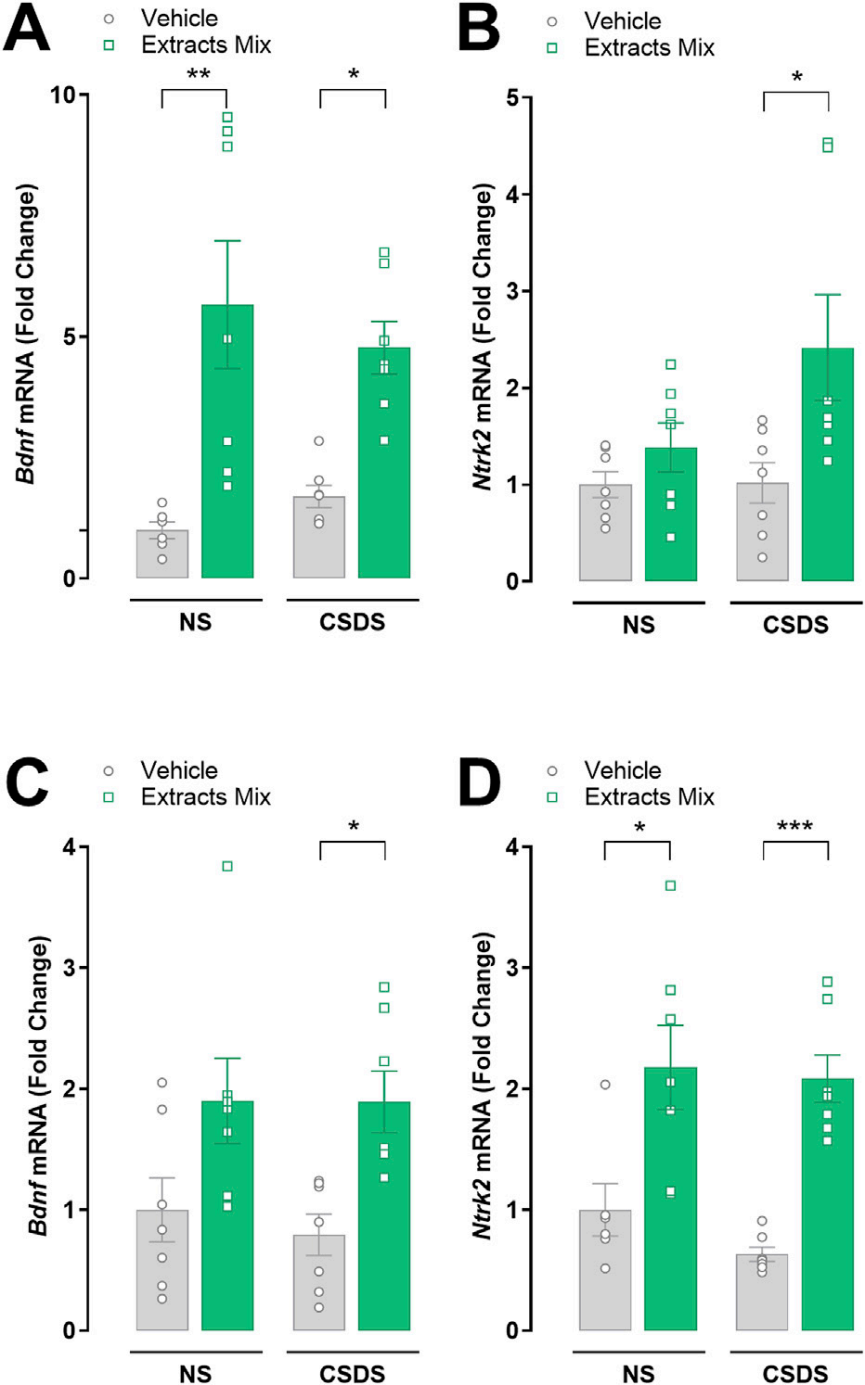
Effects of chronic treatment with extracts mix on BDNF and TrkB transcriptional expression in hippocampal **(A, B)** and cortical **(C, D)** samples. Fold changes in mRNA expression of *Bdnf* **(A, C)**, *Ntrk2* **(B, D)** genes relative to the control group determined by RT-PCR. *P < 0.05 **P < 0.01 ***P < 0.001, Two-Way ANOVA and Bonferroni’s test. N = 7–8 animals per experimental group.

Next, we studied whether stress and the extracts mix treatment influenced the transcription of the growth factor BDNF and its receptor TrkB in hippocampal and cortical samples (Figure 9). Chronic treatment with extracts mix stimulated *Bdnf* mRNA expression in the hippocampus of both stressed (*P* < 0.01) and non-stressed (*P* < 0.05) mice (Figure 9A). The same trend was observed in cortical samples; however, the increase did not reach statistical significance in non-stressed animals (Figure 9C).

Regarding *Trkb* mRNA expression, a significant increase was found in cortical samples obtained from animals treated with the extracts mix, independently of CSDS exposure (Figure 9D, *P* < 0.05 vs NS-Veh and *P* < 0.001 vs CSDS-Veh). A similar effect was detected in the hippocampus of stressed mice receiving the same treatment (*P* < 0.05), whereas in the non-stressed animals the small increase did not reach statistical significance (Figure 9B).

## 4 Discussion

The CSDS is a widely adopted stress protocol relying on the social conflict between congeners in which a rodent is introduced into the home cage of an older, aggressive, dominant animal which quickly attacks the intruder forcing it to subordination. Subsequently, animals are physically separated to avoid harm, but the visual and olfactory exposure to the stressor remains. The combination of physical and emotional aspects causes long-lasting consequences on animals’ behaviour without signs of habituation (Henriques-Alves and Queiroz, 2015). Moreover, the CSDS protocol shows also an important translational value, as the pattern of repetitive attacks inflicted by the dominant mouse on their congeners closely resembles human bullying. In fact, bullies often have intentional, repetitive, and persistent aggressive behaviours towards individuals perceived as less powerful, generating on them a negative emotional state (Rani et al., 2021; Costa et al., 2022; Björkqvist, 2001). Consequently, victims of bullying tend to have poor academic and work performance and increased risk for developing psychiatric disorders (Catone et al., 2017; Wolke and Lereya, 2015). In keeping with earlier reports (Golden et al., 2011; Rani et al., 2021; Costa et al., 2022), we found that mice treated with vehicle and exposed to 10-days of CSDS spent less time interacting with a social target, which is considered a maladaptive response modelling social withdrawal, because defeated mice avoid all mice, including those derived from their own background (Berton et al., 2006). Interestingly, repeated daily treatment with *C. asiatica*, *E. purpurea* and *Z. officinale* standardized extracts largely prevented such effect. In fact, in our study, only 25% of the treated animals exposed to the CSDS protocol exhibited the social avoidance behaviour, indicating that botanical drugs extracts treatment help animals to cope with enduring stress exposure and thus promotes a pro-resilient phenotype. Next we assessed other domains known to be markedly affected by chronic stress exposure, such as pain (Sawicki et al., 2019; Pagliusi et al., 2018), cognition (Monleón et al., 2016; Rani et al., 2021; Costa et al., 2022) and mood (Iñiguez et al., 2016).

Chronic treatment with the mix of botanical drugs extracts counteracted CSDS-induced mechanical and thermal allodynia as well as the increased expression of pain-related genes as *Scl1a3* (Sung et al., 2003; Weng et al., 2006) and *Gfap* (Sweitzer et al., 2001; Ridet et al., 1997) in the spinal cord. We observed that the same treatment also prevented the stress-induced cognitive deficits. These data are in line with our previous evidence showing the anti-hypersensitivity and procognitive effects of this treatment in a mouse model of neuroinflammation (Micheli et al., 2022). Moreover, there are several studies reporting the analgesic properties of purified metabolites obtained from the species used here, such as 8-shogaol and zingiberene from *Z. officinale* (Cheng et al., 2024; Borgonetti et al., 2023), alkylamides and polyphenols from *E. purpurea* (Micheli et al., 2023) and asiatic acid from *C. asiatica* (Huang et al., 2011). The procognitive properties of these species are also abundantly reported in the literature, in particular for *C. asiatica*, whose memory enhancing properties have been described in adult (Sbrini et al., 2020) and aged animals (Gray et al., 2018), as well as in animal models of Alzheimer’s disease (Veerendra Kumar and Gupta, 2003). The presence of these metabolites in our extract mix may have contributed to the effects observed in our experiments preventing short-term memory recognition memory deficits.

Behavioural despair models, such as the forced swimming and tail suspension tests, are widely used to assess potential antidepressant-like effects. Their principle is similar: mice are exposed to an inescapable, short-duration, moderately stressful condition, and the time they spend responding actively (escape-related behaviours) vs. passively (immobility) is measured as an index of behavioural despair/helplessness. Treatment with antidepressant-like drugs stimulates escape-related behaviours consequently reducing the time animals spend immobile (Cryan et al., 2005; Nestler and Hyman, 2010; Castagné et al., 2011). Interestingly, we found that the chronic treatment with the mix of extracts reduced the time the animals spent immobile in the tail suspension test. Such effect was observed in both stressed and non-stressed groups. Therefore, our results suggest that the extract mix possesses antidepressant-like effects independently of stress exposure. The results obtained during the behavioural evaluations fit well with the *ex vivo* analysis, as the treatment with the extracts mix stimulated cortical and hippocampal translational expression of BDNF and its receptor TrkB. BDNF is a member of the neurotrophin family most abundantly expressed in the CNS and by binding and activating the TrkB receptor, it acts as a key regulator of neuronal growth and survival, as well as of neuronal plasticity required to adapt to life events (Colucci-D'Amato et al., 2020). In our study, CSDS did not affect BDNF/TrkB brain expression as reported for other stress protocols (Huang et al., 2022; Miao et al., 2020). Nonetheless the treatment with extract mix augmented BDNF/TrkB mRNA, independently of stress exposure, a result that may explain the potential antidepressant-like effects observed in our experiments. Increased BDNF production and signalling through TrkB has a major role in mood-improving actions of classical antidepressants including classical tricyclic, monoamine oxidase inhibitors, serotonin-selective reuptake inhibitors and others (Casarotto et al., 2021; Castrén and Monteggia, 2021). More recently it was discovered that several antidepressants can directly bind TrkB and allosterically stimulate TrkB signaling (Casarotto et al., 2021), which have been hypothesized as the mechanism underpinning the effects of fast-acting antidepressants such as ketamine, lysergic acid diethylamide (LSD) and psilocin (Moliner et al., 2023; Kim et al., 2024). In this regard, previous studies demonstrated that treatment with bioactive metabolites present in the extracts mix such as chicoric acid and echinacoside from *E. purpurea* (Chuang et al., 2022; Kour and Bani, 2011), asiaticoside and asiatic acid from *C. asiatica* (Lin et al., 2013; Wang et al., 2020) and 6-shoagoal from *Z. officinale* (Afzal et al., 2022) elicits antidepressant effects and modulates brain BNDF levels.

Another important finding of this study is that the treatment with extracts mix attenuated the neuroinflammatory profile observed following CSDS exposure. The CNS and peripheral immune system are in constant communication and influence how each other respond to a challenge. Stressors, particularly of a social nature, are well known for their ability to impact the immune system. Transient neuroinflammation induced by bouts of social stress is not inherently detrimental, but adaptive in the acute phase (Hollis et al., 2022). However, prolonged or severe stress exposure causes elevated and sustained proinflammatory signalling in the CNS which is supposedly linked with stress-related psychiatric disorders (Weber et al., 2017). We found that mice exposed to CSDS show a neuroinflammatory profile in stress-sensitive brain regions, such as hippocampus and frontal cortex characterized by increased proinflammatory (IL-1β, TNF-α, IL-6) and reduced anti-inflammatory (IL-4 and IL-10) cytokines gene expression. Another evidence of the strong association between neuroinflammation and stress susceptibility is that CSDS-induced deleterious effects can be blocked or reversed by inhibiting the inflammatory system with different approaches, such as infusion of the IL-1β receptor antagonist (Wood et al., 2015; Li et al., 2023), IL-6 neutralizing antibodies (Hodes et al., 2014), or minocycline, a robust inhibitor of microglial activation (McKim et al., 2016). Chronic treatment with the extracts mix normalized the alterations induced by stress, suggesting that modulating neuroinflammation related processes may play a key role on the observed effects. These results are in line with previous publications in which anti-inflammatory properties of the extracts and of the isolated metabolites have been discussed (Cheng et al., 2024; Grzanna et al., 2005; Micheli et al., 2022; Sasmita et al., 2018).

Another stress-induced alteration closely associated with neuroinflammation, is the impairment of the blood-brain barrier (BBB) integrity. It is reported that the accumulation of proinflammatory cytokines causes BBB leakage, promoting the infiltration of different immune cells, further potentiating neuroinflammation which in turn profoundly affects behaviour (Takata et al., 2021). In our hands, no significant differences emerged between non-stressed and stressed animals treated with vehicle. However, in animals chronically treated with extracts mix and submitted to CSDS protocol we found increased mRNA levels of a series of markers related to the BBB integrity in cortical samples. Further investigation is required to understand the implication of these observations on the preventive effects induced by the extracts mix.

One limitation of our study is the lack of comprehensive safety/tolerability data, which are particularly relevant for long-term treatment with botanical drugs preparations (Izzo et al., 2016). Even though we did not specifically address toxicological aspects, no evidence of malaise was observed during the experiments, as confirmed by no differences among experimental groups in terms of food consumption and body weight gain. Moreover, no differences in terms of distance travelled or number of falls were observed in the open field and rotarod tests, respectively, suggesting that neither stress nor the treatment had a major impact on motor activity and coordination. *Zingiber officinale, C. asiatica,* and *E. purpurea* extracts have been used in traditional medicine for a long time, and phyto-pharmaceutical preparations containing extracts of these plants are currently used in clinics to treat different ailments. Consistently, phase I and II clinical trials demonstrate that extracts obtained from these plants are well tolerated (Martins et al., 2019; Wright et al., 2022; Ogal et al., 2021). However, extrapolating such safety conclusions to the present report must be done prudently since here we evaluated the effects of co-administration of the extracts. Such limitations highlight the importance of further research aiming at evaluating the tolerability and toxicological aspects associated with extract mix treatment within the dose range employed in our study to provide more robust considerations relative to their use as a therapeutic strategy.

## 5 Conclusion

Prolonged exposure to stress causes a constellation of alterations which increases the risk for the onset of several diseases. In this study, we demonstrated that repeated daily treatment with the combination of standardized extracts obtained from *C. asiatica* (200 mg/kg, i.p.), *E. purpurea* (20 mg/kg, i.p.) and *Z. officinale* (150 mg/kg, i.p.) had two main effects overall: it prevented the maladaptive responses induced by chronic stress (social avoidance, short-term recognition memory impairment and reduced thermal and mechanical allodynia) and provided potential efficacy as antidepressant independently of stress exposure. *Ex vivo* gene expression analysis suggests that the modulation of neurotrophins, cytokines and pain related markers in the hippocampus, frontal cortex and spinal cord may be the mechanisms underpinning the extracts-induced effects. The extracts mix used is rich in bioactive metabolites acting at multiple targets and holding intrinsic pharmacological properties that potentially contributed to the overall observed effects. Taken together these results suggest that standardized botanical drug extracts may be a promising strategy to promote resilience preventing the deleterious effects related to prolonged stress exposure.

## Data Availability

The raw data supporting the conclusions of this article will be made available by the authors, without undue reservation.
